# Intrinsic and extrinsic motivation is associated with computer-based auditory training uptake, engagement, and adherence for people with hearing loss

**DOI:** 10.3389/fpsyg.2015.01067

**Published:** 2015-08-06

**Authors:** Helen Henshaw, Abby McCormack, Melanie A. Ferguson

**Affiliations:** ^1^Otology and Hearing Group, National Institute for Health Research Nottingham Hearing Biomedical Research Unit, Division of Clinical Neuroscience, School of Medicine, University of NottinghamNottingham, UK; ^2^Nottingham University Hospitals NHS TrustNottingham, UK

**Keywords:** auditory training, motivation, engagement, adherence, hearing loss, sensorineural, Self-Determination Theory

## Abstract

Hearing aid intervention typically occurs after significant delay, or not at all, resulting in an unmet need for many people with hearing loss. Computer-based auditory training (CBAT) may provide generalized benefits to real-world listening, particularly in adverse listening conditions, and can be conveniently delivered in the home environment. Yet as with any intervention, adherence to CBAT is critical to its success. The main aim of this investigation was to explore motivations for uptake, engagement and adherence with home-delivered CBAT in a randomized controlled trial of adults with mild sensorineural hearing loss (SNHL), with a view to informing future CBAT development. A secondary aim examined perceived benefits of CBAT. Participants (*n* = 44, 50–74 years olds with mild SNHL who did not have hearing aids) completed a 4-week program of phoneme discrimination CBAT at home. Participants' experiences of CBAT were captured using a post-training questionnaire (*n* = 44) and two focus groups (*n* = 5 per group). A mixed-methods approach examined participants' experiences with the intervention, the usability and desirability of the CBAT software, and participants' motivations for CBAT uptake, engagement and adherence. Self-Determination Theory (SDT) was used as a theoretical framework for the interpretation of results. Participants found the CBAT intervention easy to use, interesting and enjoyable. Initial participation in the study was associated with extrinsic motivation (e.g., hearing difficulties). Engagement and adherence with CBAT was influenced by intrinsic (e.g., a desire to achieve higher scores), and extrinsic (e.g., to help others with hearing loss) motivations. Perceived post-training benefits included better concentration and attention leading to improved listening. CBAT also prompted further help-seeking behaviors for some individuals. We see this as an important first-step for informing future theory-driven development of effective CBAT interventions.

## Introduction

In 2008, the World Health Organization estimated that over 360 million people worldwide had a disabling hearing loss. These figures are expected to rise substantially in the future due to aging of the global population (World Health Organization, 2008). Hearing loss currently affects more than 10 million adults in the UK alone (Action on Hearing Loss, 2011), which corresponds to approximately one in six of the population. The most common management strategy for hearing loss is the provision of hearing aids, which primarily help restore audibility. However, just one in three people who could benefit from hearing aids in the UK actually have them (Davis et al., 2007), resulting in an unmet need for an estimated four million people. For those who do seek intervention, this process takes an average of 10 years, which may in part be related to the fact that 47% of those individuals who report hearing difficulties to their family doctor fail to receive an onward referral to Audiology services (Davis et al., 2007). A scoping review of the literature suggests that of those individuals who do seek audiological intervention and are fitted with hearing aids, between 4 and 24% choose not to use them (McCormack and Fortnum, 2013). In addition, many choose not to use them regularly (Whitmer et al., 2014). Untreated hearing loss can lead to numerous social and emotional issues, including; difficulties with work, social withdrawal, isolation, and depression (Davis et al., 2007). In addition, recent findings from a large cohort study identified an association between hearing loss and incident dementia, whereby the risk increases with the degree of impairment (Lin et al., 2011a,b). Auditory (re)habilitation is however, much wider than the provision of hearing aids alone (Ferguson and Henshaw, 2015b).

One of the most common complaints of people with hearing loss is difficulty listening to speech in the presence of distractors, such as competing talkers or background noise (e.g., Pichora-Fuller and Singh, 2006). In recent years, the role of top-down (cognitive) processes in listening have been subject to rigorous examination that is sufficient to warrant its own field of research, Cognitive Hearing Science (Arlinger et al., 2009; Rönnberg et al., 2011). Speech in noise performance is associated with cognition, and the role of cognition becomes increasingly important as the complexity of the listening task increases (Heinrich et al., 2015). Auditory training is one type of intervention for those with hearing loss, which can be described as teaching the brain to listen through active engagement with sounds (Schow and Nerbonne, 2006). Auditory training is designed to improve an individuals' use of their residual hearing through repeated listening practice (Tye-Murray et al., 2012). Both basic and applied research has identified top-down influences of auditory training (Amitay et al., 2006; Sweetow and Henderson Sabes, 2006; Pichora-Fuller and Levitt, 2012; Anderson et al., 2013). For example, Amitay et al. (2006) show that participants are better able to discriminate tone frequency after training on a task that uses identical frequency stimuli. The authors attribute this post-training improvement to both bottom-up and top-down influences, including selective attention and arousal. Evidence from our own research takes this further by suggesting that the benefits of auditory training may be primarily driven by top–down mechanisms, and that these benefits are most evident for challenging listening conditions that index executive processes such as the updating of working memory and attentional control (Ferguson and Henshaw, 2015a,b). These conclusions are based on the results of a randomized controlled trial of 44 adults with mild bilateral sensorineural hearing loss (SNHL) (Ferguson et al., 2014). Post-training outcomes showed significant improvements in divided attention, updating of working memory, and self-reported hearing abilities in a challenging listening condition (“talking with several people in a group”) for the trained group, with no improvements for the control group, following a 4-week at-home phoneme discrimination training program (total training time = 6 h). There were no significant improvements shown for a sentence in noise perception task. However, a second study assessing cognitively demanding listening tasks showed a significant improvement for a two-competing talker task (Modified Coordinate Response Measure) of 2.3 dB signal to noise ratio (SNR), following just 3.5 h phoneme discrimination in noise training (Henshaw and Ferguson, 2014).

Yet, as with any intervention, auditory training can only ever be effective if adhered to. In a recent systematic review of 13 articles assessing the efficacy of individual computer-based auditory training (CBAT) for people with hearing loss (Henshaw and Ferguson, 2013), compliance with CBAT was reported in less than half (6/13) of the studies. Where it was reported, compliance rates were high for both laboratory-based (81%) and home-based (73–100%) interventions. However, variation in the definition of training compliance was a particular issue highlighted by the systematic review, with authors often choosing to report either the proportion of participants who did not drop out of the study (i.e., study completion), or the proportion of participants who completed the recommended amount of training (intervention compliance). Sweetow and Henderson Sabes (2006) argued that for widespread use in adults with hearing loss, CBAT programs should be easy, fun, and rewarding, incorporating both top-down and bottom-up approaches to auditory learning. However, data collected from over 3000 patients in routine clinical practice who used the Listening and Communication Enhancement (LACE) CBAT program showed compliance rates of >30%, where compliance was defined as completion of 10 or more of the 20 recommended training sessions (Sweetow, 2009). In another study using LACE, 50 veterans with hearing loss who completed the 20 recommended sessions gained generalized benefits in untrained measures of rapid speech and speech understanding in noise, with no significant improvements for non-compliers (Chisolm et al., 2013). Some of the key challenges associated with auditory training adherence are thought to include; lack of recommendation by audiologists, the nature of the trained task, and the misalignment of audiologist and patient goals (Sweetow and Henderson Sabes, 2010). Nevertheless, these challenges have yet to be confirmed with evidence. In 2003, The World Health Organization placed strong emphasis on the need to differentiate the terms compliance and adherence (World Health Organization, 2003). The main difference is that adherence requires the patient's agreement to the recommendations set, whereas compliance may be more closely associated with blame.

Human behavior is the largest source of variance in health-related outcomes (Schroeder, 2007). Literature from chronic health domains suggests that individuals' motivations play a significant role in treatment adherence (Vermeire et al., 2001). Motivation controls and sustains goal-directed behaviors, with three main components; activation (the decision to initiate the behavior), persistence (continued effort toward a goal even though obstacles may exist), and intensity (the concentration and vigor that goes into pursuing a goal). For home-based training interventions that extend over a number of weeks, there are likely to be several motivational factors that impact on individuals' levels of engagement and adherence with the intervention (Sweetow and Henderson Sabes, 2010). Behavioral science offers opportunities to develop and advance digital health interventions (Pagoto and Bennett, 2013), whereby insights from health behavior psychology can improve our understanding of auditory training adherence and highlight consideration for future auditory training development (Tye-Murray et al., 2012).

**Figure 1:**
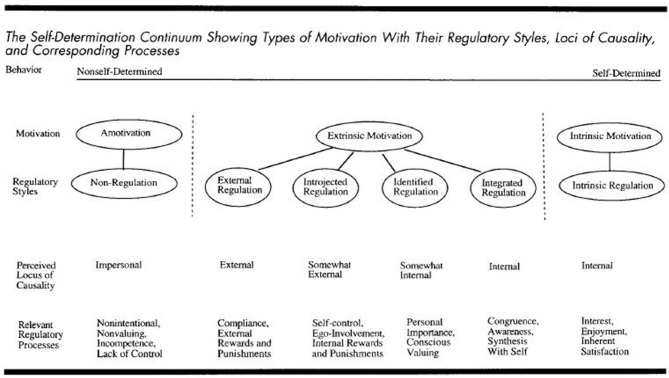
**The self-determination continuum, Ryan and Deci (2000)**. Copyright © 2000 by the American Psychological Association. Reproduced with permission. The official citation that should be used in referencing this material is Ryan and Deci (2000). The use of APA information does not imply endorsement by APA.

Self-Determination Theory (SDT; Deci and Ryan, 1985) is an approach to motivation that is concerned with supporting people's natural tendencies to behave in effective and healthy ways. SDT distinguishes between different types of motivation based on the different reasons or goals that give rise to an action. The basic distinction is between intrinsic motivation, which refers to doing something because it is inherently interesting or enjoyable, and extrinsic motivation, which refers to doing something because it leads to a separable outcome (Ryan and Deci, 2000). As such, intrinsic motivation is important for completing a task, whereas extrinsic motivation reflects acceptance of the value or utility of a task. This can be conceptualized as a self-determination continuum (Figure 1). SDT emphasizes processes through which a person internalizes health behaviors so that they may be self-determined (Ryan et al., 2008). The theory highlights three basic human psychological needs, which when satisfied yield enhanced motivation and well-being (Ryan and Deci, 2000):

- *Autonomy:* the feeling of psychological freedom or choice.
- *Competence:* perceived self-efficacy (i.e., one's belief in one's ability to succeed).
- *Relatedness:* the need to feel belongingness and connectedness with others.

SDT has previously been employed to examine individuals' motivations for hearing aid use (Ridgeway et al., 2013, 2015), and may provide a useful framework to better understand individuals' motivations for engagement and adherence to other hearing interventions, such as CBAT. Any novel insights gained from SDT may be used to inform the future development of feasible and effective CBAT interventions for people with hearing loss.

Auditory training has the potential to be a useful intervention for people with hearing loss, including hearing aid users and those who choose not to wear hearing aids, or those who have mild hearing loss and would not necessarily benefit substantially from amplification. The present study focused on adults with mild SNHL who were experiencing hearing difficulties, but had not yet sought intervention for their hearing loss. A randomized controlled trial (RCT) of 44 adults with mild SNHL examined the effects of a 4-week home-based program of CBAT on speech perception, cognition and self-reported hearing abilities (Ferguson et al., 2014). Participants completed a 4-week program of CBAT at home. There were high levels of adherence with CBAT, whereby 80% of participants (*n* = 35) completed the recommended amount of training (6 h over 4 weeks) and 75% (*n* = 33) exceeded the recommended training, with no participant drop outs (Ferguson et al., 2014). Findings showed significant post-training improvements in cognition and self-reported hearing abilities, particularly for challenging task conditions. However, it remains untested as to whether these benefits were readily perceived by the study participants.

The main aim of the present investigation was to explore participants' motivations for high levels of adherence, uptake and engagement with CBAT in this study using SDT as the theoretical framework. This was achieved through:

- a post-training feedback questionnaire, administered in the RCT,
- two post-RCT focus groups.

A secondary aim sought to qualitatively examine the perceived benefits of the CBAT intervention and compare this with the published (quantitative) behavioral RCT results (Ferguson et al., 2014).

## Materials and methods

The study was approved by the Nottingham Research Ethics Committee and Nottingham University Hospitals NHS Trust Research and Development. Signed, informed consent was obtained.

### Participants

#### Randomized controlled trial

Adult non-hearing aid users were recruited to take part in the RCT from three General Practitioner (family physician) surgeries in Nottingham, UK (see Ferguson et al., 2014 for full details of the study design, procedure, and post-training outcomes). Participants (29 male, 15 female) were aged 50–74 years old (mean = 65.3 years, *SD* = 5.7 years) with mild, symmetrical SNHL (mean hearing thresholds averaged across 0.5, 1, 2, and 4 kHz = 32.5 dB HL, *SD* = 6.0 dB HL, with a left-right difference of < 15 dB). Computer literacy ranged from “never used a computer” (*n* = 7), to “beginner” (*n* = 20), and “competent” (*n* = 17).

#### Focus groups

Ten participants from the RCT (seven male, three female) volunteered to participate in one of two focus groups (five per group). Mean age was 64.8 years (*SD* = 5.7 years), and mean better ear hearing thresholds averaged across 0.5, 1, 2, and 4 kHz = 30.4 dB HL, (*SD* = 6.1 dB HL). Participants travel expenses were paid, and they received a £10 inconvenience fee for their visit.

### Procedure

#### Randomized controlled trial

Participants were randomized to one of two groups in a randomized, quasi-crossover study design (Ferguson et al., 2014). The Immediate training group attended three test sessions (pre-training, post-training, and 4 week follow-up), the delayed training group attended four test sessions (control baseline, pre-training, post-training, and 4 week follow-up).

Participants completed a 4-week program of computer-based phoneme discrimination training at home, using a loan laptop which was specially programmed with only the CBAT (phoneme discrimination) training software. Training stimuli were 11 phoneme continua (/a/-/uh/, /b/-/d/, /d/-/g/, /e/-/a/, /er/-/or/, /i/-/e/, /l/-/r/, /m/-/n/, /s/-/sh/, /s/-/th/ and /v/-/w/), synthesized from end-points consisting of real voice recordings, delivered for 15 min/day, 6 days/week, for 4 weeks. The training was a 3-interval, 3-alternative forced choice task. During training, participants heard three phoneme sounds presented sequentially by three on-screen characters. They were then asked to select the character who made the “odd one out” phoneme sound. Participants completed two short (five-trial) familiarization demonstrations with the researcher in the laboratory prior to at-home training.

Training was delivered using software developed at the MRC Institute of Hearing Research (IHR-STAR) but with graphics designed for adult participants (Ferguson et al., 2014). Visual feedback (character waving) indicated correct responses to participants on a trial-by-trial basis. Participants were contacted once a week via telephone during the 4-week training period. This was to monitor participants' progress and to identify and resolve any technical or procedural issues with training.

Outcome measures were administered at each test session to assess participants' speech perception performance (Sentence and digit perception in noise tasks), cognition (single and divided attention, working memory), and self-reported hearing (Glasgow Hearing Aid Benefit Profile, Speech, Spatial and Qualities of Hearing) (Ferguson et al., 2014).

##### Post-training feedback questionnaire

At the post-training test session, a questionnaire (adapted from Benedek and Milner, 2002) was used to assess participants' views of the CBAT intervention and the usability and desirability of the training software. The questionnaire was administered to all RCT participants by interview at the post-training session and consisted of three sections:

- *Statements:* Participants were asked to rate whether they agreed or disagreed with 10 short statements describing their experience with the CBAT software using a five-point Likert scale (strongly agree to strongly disagree).
- *Descriptor words:* Sixty descriptor words were presented on response cards and were categorized as positive (+, *n* = 35), negative (−, *n* = 23) or could be interpreted as either positive or negative (+∕−, *n* = 2). Participants identified words that described their experience with the CBAT program and of those, participants prioritized the five words most relevant to their experience.
- *Open-ended questions:* Participants were asked three open-ended questions to assess the (i) worst, and (ii) best aspect of their experience with the training program, and (iii) any changes that would make the program more interesting, enjoyable or engaging. Content analysis (Krippendorff, 2004) was used by one researcher (HH) to develop mutually exclusive themes that identified the content of participants' responses.

#### Focus groups

Three key questions were considered in the focus groups;

1. What motivated participants to take part in the CBAT study?
2. Why did participants engage and comply with the CBAT program? And,
3. What were the perceived benefits of the CBAT program?

These questions were supplemented by additional probe questions to ensure that discussions were detailed and remained on track. The focus groups lasted 2.5 and 2 h, respectively, and were each facilitated by two researchers (MAF and HH) in a quiet room, free from distraction. The majority of questions were asked by the primary facilitator (MAF).

The focus groups were audio recorded using a high quality audio recorder and transcribed verbatim. Focus group transcripts were entered into *QSR Nvivo* (Version 8). Thematic analysis was based on guidelines by Braun and Clarke (2006). To facilitate the emergence of themes, the transcripts were read, reviewed, reread and reviewed again, by one researcher (AM), to gain familiarity with the content. Analysis began with open coding to catalog what was seen to be “going on” in the data. Themes were identified by re-visiting the codes and the data, to which they had been applied, to rethink, revise and develop higher order categories.

## Results

### Randomized controlled trial

A summary of the quantitative results from the auditory training efficacy RCT are provided below. For detailed analyses, see Ferguson et al. (2014).

*Auditory training:* For CBAT, robust phoneme discrimination learning was found for both immediate training and delayed training groups, with the largest improvements in threshold shown for phoneme pairs with the poorest initial thresholds.
*Outcome assessment:* The immediate training group showed significant improvements in self-reported hearing, divided attention, and working memory. However, training did not result in consistent improvements in speech perception in noise. There was no evidence of any significant improvements in performance on any of the outcomes for the delayed training (control) group.
*Follow-up assessment:* Retention of benefits at 4 weeks post-training for the immediate training group was shown for phoneme discrimination, divided attention, working memory, and self-report of hearing disability.

#### Aim 1: exploring motivations for CBAT uptake, engagement, and adherence

Data from the questionnaires and focus groups are interpreted as being representative of intrinsic or extrinsic motivation according to SDT (Ryan and Deci, 2000), based on the Self-Determination Continuum (Figure 1).

##### Post-training feedback questionnaire Statements

Frequencies of participants' responses to the 10 statements are summarized in Table 1.

**Table 1:** **Number and percentage of total participants (*n* = 44) responding to statements about their experiences with the CBAT intervention. SDT = Self Determination Theory**.

**Statement**	**SDT Motivation type**	**Strongly disagree**	**Disagree**	**Neither agree nor disagree**	**Agree**	**Strongly agree**
The training program held my interest	Intrinsic	0 (0.0%)	4 (9.1%)	5 (11.4%)	23 (52.3%)	12 (27.3%)
I enjoyed training with the program	Intrinsic	0 (0.0%)	1 (2.3%)	5 (11.4%)	20 (45.5%)	18 (40.9%)
I found my attention on the training program wandered during the session	Intrinsic	16 (36.4%)	16 (36.4%)	5 (11.4%)	6 (13.6%)	1 (2.3%)
I understood what to do when using the training program	Intrinsic	0 (0.0%)	0 (0.0%)	1 (2.3%)	18 (40.9%)	24 (54.5%)
I would never use this training program again	Intrinsic	24 (54.5%)	15 (34.1%)	2 (4.5%)	3 (6.8%)	0 (0.0%)
I found the training program difficult to use	Intrinsic	27 (61.4%)	15 (34.1%)	1 (2.3%)	0 (0.0%)	1 (2.3%)
The training program did not let me know how I was doing	Intrinsic	11 (25.0%)	15 (34.1%)	4 (9.1%)	10 (22.7%)	4 (9.1%)
I felt motivated to use the training program regularly	Extrinsic	0 (0.0%)	2 (4.5%)	8 (18.2%)	21 (47.7%)	13 (29.5%)
Doing the training made me more aware of my hearing	Extrinsic	0 (0.0%)	3 (6.8%)	6 (13.6%)	19 (43.2%)	16 (36.4%)
I did the training because it might make my hearing better	Extrinsic	1 (2.3%)	7 (15.9%)	5 (11.4%)	17 (38.6%)	14 (31.8%)

##### Intrinsic motivation

The majority of participants agreed that the CBAT intervention was both interesting and enjoyable, suggesting there was intrinsic motivation to undertake training. Most agreed or strongly agreed with the statements “The training program held my interest” (*n* = 35, 79.5%) and “I enjoyed training with the program” (*n* = 38, 86.4%), which is indicative of participants acting of their own free will (autonomy). There was little agreement shown for “I found my attention on the training program wandered during the session” (*n* = 7, 15.9%), suggesting that those participants were actively engaged with the CBAT. Finally, low agreement with the item “I found the training program difficult to use” (*n* = 1, 2.3%) and high agreement with “I understood what to do when using the training program” (*n* = 42, 95.5%) demonstrates competence in participants' ability to undertake CBAT. Only, three participants (6.8%) agreed with the statement “I would never use this training program again.”

##### Extrinsic motivation

The majority of participants (*n* = 34, 77.3%) agreed or strongly agreed with the statement “I felt motivated to use the training program regularly.” Although the reasons for this motivation cannot be inferred from responses to this question alone, responses to item “I did the training because it might make my hearing better” (*n* = 31, 70.5%) suggested that there were extrinsic motivations for participating in the training. The majority of participants also agreed with the statement “Doing the training made me more aware of my hearing” (*n* = 35, 79.5%).

###### Descriptor words

Participants' descriptor word selections are presented in Table 2, ranked in order of frequency. Participants selected an average of 22.48 (*SD* = 7.28) words to describe their experiences with the CBAT. All descriptor words were selected at least twice across all participants. Of the first 30 words in the table, 28 are positive (93.3%) and only one is negative (3.3%), with an average of 26.5 (*SD* = 12.1) participant selections per item. Of the last 30 words in the table, seven are positive (23.3%), and 22 (73%) are negative, with an average of 6.2 (*SD* = 3.4) participant selections per item.

**Table 2:** **Frequency of participants' word choices to describe their experience with the CBAT intervention**.

**Rank**	**Descriptor word**	**Positive (+) negative (−) or ambiguous (+∕−)**,	**Frequency of selection by *n* = 44 participants**
1	Easy to use	+	43
2	Straightforward	+	40
3	Organized	+	38
4	Rewarding	+	36
5	Accessible	+	36
6	Valuable	+	34
7	Motivating	+	34
8	Fun	+	34
9	Usable	+	32
10	Repetitive	–	31
11	Consistent	+	31
12	Useful	+	30
13	Relevant	+	30
14	Efficient	+	30
15	Simplistic	+/–	29
16	Familiar	+	26
17	Stimulating	+	25
18	Appealing	+	25
19	Reliable	+	22
20	High quality	+	20
21	Trustworthy	+	20
22	Predictable	+	19
23	Attractive	+	19
24	Comprehensive	+	18
25	Personal	+	18
26	Connected	+	18
27	Inviting	+	16
28	Fast	+	14
29	Desirable	+	13
30	Flexible	+	13
31	Fresh	+	13
32	Empowering	+	12
33	Exciting	+	11
34	Frustrating	–	10
35	Tedious	–	10
36	Collaborative	+	10
37	Boring	–	9
38	Sophisticated	+	9
39	Busy	–	9
40	Unpredictable	–	9
41	Time consuming	–	8
42	Customizable	+	8
43	Rigid	–	7
44	Unconventional	+/–	7
45	Slow	–	6
46	Confusing	–	5
47	Uncontrollable	–	5
48	Dull	–	4
49	Intimidating	–	4
50	Overwhelming	–	4
51	Inconsistent	–	3
52	Stressful	–	3
53	Complex	–	3
54	Gets in the way	–	3
55	Time saving	+	3
56	Hard to use	–	2
57	Not valuable	–	2
58	Overbearing	–	2
59	Patronizing	–	2
60	Too technical	–	2

The five most frequently selected words “Easy to use,” “Straightforward,” “Organized,” “Rewarding and accessible” are intrinsic in nature, and reflect autonomy and competence (i.e., participants are willing and able to complete the CBAT). Word selections such as “Valuable” and “Relevant” on the other hand suggest extrinsic motivations, whereby participants are identifying with and consciously valuing the CBAT intervention.

**Figure 2:**
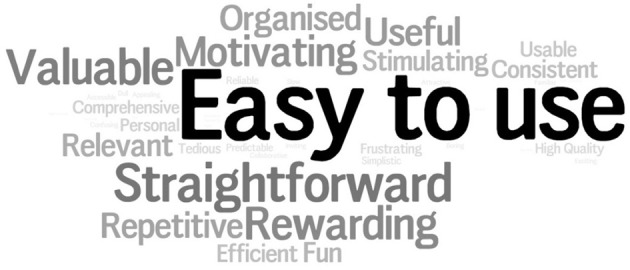
**Word cloud to show participantŠs top five word choices describing their experience with the auditory training software**.

Frequency of selection for participants' top five prioritized descriptor words to describe their experience with the auditory training software is illustrated in a word cloud (Figure 2), where words with the greatest frequency of selection appear larger and darker than those words that were less frequently selected. “*Easy to use*” (intrinsic motivation) was by far the most frequently selected by participants as one of the top-five descriptors to explain their experiences with the CBAT software (28/44 participants, 63.6%). Other frequently prioritized words, selected by at least a quarter of all participants, included *Straightforward* (intrinsic; *n* = 15, 34.1%), *Valuable* (extrinsic; *n* = 14, 31.8%), *Rewarding* (extrinsic; *n* = 13, 29.5%), *Motivating* (extrinsic; *n* = 12, 27.3%) and *Useful* (extrinsic; *n* = 11, 25.0%).

###### Open-ended questions

**1. What was the best aspect(s) of your experience with the training program?**

Answers to this question were grouped into seven main themes (italicized). Themes are reported here in the order most commonly referred to, and grouped according to intrinsic and extrinsic motivations.

##### Intrinsic motivation

*An easy and enjoyable task:* was reported by 12 participants (27.2%). *Sense of achievement associated with completing the training:* reported by four participants (9.0%).

##### Extrinsic motivation

*Increased awareness of hearing or hearing difficulties:* reported by eight participants (18.2%). *To aid research*: was offered as a response by one participant (2.3%).

In addition, a number of participants provided unprompted accounts of perceived benefits of the CBAT intervention, including; *improved listening, concentration, or attention post-training:* 10 participants (22.7%) and *Improved PC literacy* or *a desire to further improving their PC literacy*: reported by two participants (4.5%).

**2. What was the worst aspect(s) of your experience with the CBAT program?**

Responses to this question were grouped into six main themes:

##### Intrinsic motivation

*Technical issues with training hardware or software:* reported by 17 participants (38.6%). *Training tasks were repetitive or boring:* reported by seven participants (15.9%). *Feedback in the training software was not satisfactory:* five participants (11.4%) felt that the feedback did not always reflect how they perceived they were performing. *Performance on the training task:* four participants (9.1%) reported they were unhappy with their own performance in the CBAT intervention.

##### Extrinsic motivation

*Practical issues with training:* Eight participants (18.2%) reported issues such as finding time to train, or setting up and putting away a laptop computer. Finally, *Lack of experience with computers*: reported by two participants (4.5%).

**3. What would you change to make the training program more interesting, enjoyable or engaging?**

Responses were grouped into five main themes. All responses related to the nature of the CBAT software itself and are therefore interpreted as most relevant to intrinsic motivation.

##### Intrinsic motivation

*Software changes*: Ten participants (22.7%) reported the software could be improved, for example, changes to the feedback provided or the adaptive nature of the training games. Nine participants (20.5%) suggested *improved graphics*, seven participants (15.9%) wanted to see *changes to the sounds*, three participants (6.8%) suggested *more game-play in training tasks*, and three participants (6.8%) suggested *more variety* in the CBAT software.

### Focus groups

Thematic analysis of focus group transcripts provided main themes and sub-themes for each of the three main research questions, summarized in Table 3.

**Table 3:** **Main categories and sub themes from thematic analysis of focus group transcripts**.

**Question**	**Main categories**	**Sub-themes**
1. What motivated participants to take part in the CBAT study?	Hearing difficulties (extrinsic motivation)	Difficulty hearing in certain situations
		Relatives/friends commenting on their hearing difficulties
		Wanted to find out more about their hearing difficulties
		Curious about hearing loss
		A desire to improve their listening abilities
		Received invitation letter from family physician
2. Why did participants engage and comply with the CBAT program?	A sense of achievement (intrinsic motivation)	Desire to beat previous scores
		Seeing the training through to the end
	A desire and capacity to help others (extrinsic motivation)	To help others by taking part in research
		Having spare time to fill
3. What were the perceived benefits of the CBAT program?	Increased concentration, attention and focus in everyday listening	Improved listening skills
		Strategies for listening
	Encouraged further help-seeking behaviors	Seeking further hearing intervention

**1. What motivated participants to take part in the auditory training study?**

Participants were extrinsically motivated to take part in the CBAT study as a result of their *hearing difficulties*. Participants reported that they took part in the study either because they had noticed difficulty hearing in certain situations, or other people had commented on their hearing difficulties.

Participants talked about how their families had encouraged them to seek help for their hearing difficulties, and this prompted them to take part in the study:

“*The family's been on at me for a long time now about me hearing and so I thought, yeah, go for it.”*

Some participants had noticed their hearing difficulties themselves, and this made them to want to find out more about their hearing levels:

“*I was concerned about my hearing, especially with the grandchildren I couldn't always hear what they were saying and I didn't want to end up like my mum.”*

Some participants were not sure if their hearing was bad enough to require further attention, and reported wanting to take part in the research to “*catch it quick”* and to see if it would help before their hearing deteriorated further:

“*Well I'm not sure whether I'm deaf or not. You know how you are because you're on the borderline.”*

The invitation (received via their family physician) motivated some participants to take part in the study because they wanted to find out more about their hearing:

“*I knew that I had got some impairment with my hearing anyway because I keep getting this…ay? What? I thought there's something wrong here that's not quite right and as I was thinking about that, the letter came saying, would you like to take part. I thought, that's timely, yes please.”*

**2. Why did participants engage with and adhere to the auditory training program?**

Participants were both intrinsically and extrinsically motivated to engage in and adhere to the CBAT intervention. Intrinsic motivation to engage and adhere to the intervention was governed by participants' *sense of achievement* associated with on-task improvement and completion of the CBAT program.

Participants reported a challenging element to the training. They reported an intrinsic desire to beat their scores each session and this motivated them to continue:

“*I was trying to beat the other score, and I thought, yeah, I'm going to get it this time.”*“*Yes, every time I sat down, I wanted to beat the next one.”*

Participants wanted to beat their own scores as there was a sense that if they were to improve their scores then their hearing might be improving. Therefore, an extrinsic motivation leading to adherence with training was a *desire to improve their listening abilities*:

“*I was trying to do better every day and thinking, I'm going to get all these right, and then the first couple seemed quite easy and then it seemed to get really, really hard but it just made me want to do better, really, every time.”*

Participants were intrinsically motivated by the sense of achievement gained from completing the intervention program:

“*Oddly, like a sense of achievement, to actually complete the course, if you like.”*

There was also a sense of commitment among the participants. Once they had said they would do something they wanted to see it through to the end:

“*Well, it's the sort of thing that we've set out on a course of action, like [focus group participant] was saying. He [focus group participant] likes to finish things he has set out to do.”*

A secondary theme was extrinsic in nature, *the desire and capacity to help others*. Participants commented that they completed the training because they wanted to be able to help other people with hearing difficulties. They felt that if the training worked then they might be able to recommend it to other people who might benefit from it:

“*Another reason I started in the first place was the fact that I wanted to help others, you know…Let's go and see what this is about.”*

Additionally, some participants reported a desire to aid research, and all participants reported having spare time to fill, particularly those who were retired from work:

“*I thought well it's worth doing, it's worth looking at, seeing as I've got the time…obviously retired, and said, I've got the time to do this, let me do it now.”*

#### Aim 2: examining the perceived benefits of CBAT

**3. What were the perceived benefits of the auditory training program?**

The dominant theme was *increased concentration, attention and focus in everyday listening*. All but one participant reported that the training made them concentrate more:

“*…It [the training] made me concentrate more, it certainly did.”*

“*I think it just made me aware that if I do want to hear what's going off, I've got to pay attention and focus more than I used to.”*

Consequently the improved concentration and attention was associated with improved listening:

“*Yes, it does make you concentrate and think—when you are concentrating you can hear more.”*

Through increased concentration, participants reported post-training that they had developed better strategies for listening, such as “*looking at peoples' lips”*; “*people watching;”* and concentrating on the main conversation “*rather than trying to listen to three conversations*.” This theme mirrored reports of *improved listening, concentration, or attention post-training*, offered by 10 participants in the post-training questionnaire.

A secondary theme identified in the focus groups was that training *encouraged further help-seeking behaviors*:

“*I think the primary thing is identifying that there is a problem in the first place. We have, and so we have got the wherewithal to actually do something about it, which your program is good at*…”

Furthermore, participants thought CBAT may have the same effect on others by prompting them to seek further help:

“*…if [after being provided with training] they think they have got a problem, that would enhance them or encourage them to go for further tests.”*

After taking part in the CBAT study, two focus group participants had since been fitted with hearing aids. One of those individuals described CBAT as a stepping-stone to seeking further intervention:

“*From the point of view of this training, I sort of looked at it as a sort of middle ground. It, I feel that it helps me, but then I subsequently needed them [hearing aids] to help me a bit more.”*

## Discussion

The primary aim of this investigation was to examine motivations to undertake a program of home-delivered CBAT to improve listening for adults with mild hearing loss. Self-Determination Theory (SDT; Deci and Ryan, 1985) was adopted as a theoretical framework by which to interpret motivations for initial participation in the study (uptake), engagement and adherence with the CBAT intervention using data from a post-training questionnaire and two focus groups. A secondary aim was to examine the perceived benefits of CBAT to compare with the published behavioral outcomes of this study (Ferguson et al., 2014).

### Motivations for CBAT uptake, engagement, and adherence

Results from the present research showed different contributions of intrinsic and extrinsic motivation for participants' uptake, and engagement and adherence, with CBAT. The main theme explaining participants' motivations for initial participation in the study (CBAT uptake) identified from the focus groups was participants' hearing difficulties (extrinsic motivation). Sub-themes included identification of hearing difficulties by relatives or friends, participants' desire to improve their listening, and the invitation into the study being received via their family physician. These results showed that participants were extrinsically motivated to take part in the study and to try CBAT in an attempt to address their hearing difficulties.

Engagement and adherence with CBAT was shown to be influenced by both intrinsic and extrinsic motivation. Descriptors of their experiences with the intervention from the post-training questionnaires was highly positive in nature, with participants' selecting positive descriptor words more frequently than negative words. Responses to the statements showed that participants found the CBAT easy to use, suggesting competence in their ability to undertake CBAT. This was the case for the vast majority of participants in the study despite a wide range of computer skills, with seven participants having never used a computer before. In addition, participants agreed that the intervention was both interesting and enjoyable. Based on SDT, this suggests that participants were intrinsically motivated to engage and comply with the intervention, and demonstrates a level of autonomy for the task. It has been argued that competence must accompany autonomy in order for individuals to see their behaviors as self-determined by intrinsic motivation (Reeve, 1996), and future CBAT programs may benefit from addressing this specifically in their design.

Results from the focus groups suggested that on a day-to-day basis, participants in the study were intrinsically motivated to adhere to training in an attempt to beat their previous scores. In the long term, participants were committed to seeing the training intervention through to the end for the inherent satisfaction associated with program completion. A secondary theme associated with CBAT adherence was the desire and capacity to help others (extrinsic motivation). Participants believed that by completing the intervention they may help other people with hearing difficulties.

### Perceived benefits of the CBAT intervention

For the open-ended questions in the post-training questionnaire, almost a quarter of participants provided unprompted reports of improved listening, concentration, or attention post-training. Furthermore, one of the main themes explaining perceived benefits of training from thematic analysis of focus group transcripts was *increased concentration, attention and focus in everyday listening*. Focus group participants also reported that the CBAT enabled them to develop better strategies for listening, such as concentrating on the main conversation rather than trying to listen to multiple conversations at once. In the main RCT, improvements were shown for behavioral measures of complex cognition (working memory updating and divided attention), and for self-reported hearing in a group situation (Ferguson et al., 2014).

Focus group participants reported that the CBAT made them more aware of their hearing difficulties, and some participants said that taking part in the training program encouraged them to seek further intervention to address their hearing difficulties. This suggests that CBAT may act as an important stepping stone toward further intervention or help-seeking behaviors for some individuals with hearing loss.

### Future directions

“To be motivated means *to be moved* to do something” (Ryan and Deci, 2000), but evidence suggests that the maintenance and enhancement of human motivation requires supportive conditions. Although adherence was high in this study, there is evidence to suggest that adherence may be up to 50% lower in real-world clinical application (Sweetow, 2009). It is therefore important to better understand CBAT adherence in a research setting, so that CBAT interventions stand the best chance for high rates of adherence outside of the research environment.

Using SDT as a theoretical framework, we present a number of considerations for the development of future CBAT that may facilitate both intrinsic and extrinsic motivations for uptake, engagement and adherence.

#### Intrinsic motivation

In the present study, when asked about the changes they would change to make the training program more interesting, enjoyable or engaging, questionnaire respondents reported a number of software developments, including increased gameplay. Gameplay has been shown to promote enjoyment and adherence with interventions in health and education domains (e.g., Nilsson et al., 2009; Papastergiou, 2009), and this has previously been examined within ENT, using gameplay to CBAT interventions for tinnitus (Hoare et al., 2014). One approach to adequately address these software considerations would be to involve the target population themselves in the design of CBAT interventions. Collaboratively involving the end-user in the development of digital and eHealth interventions that target behavior change ensures material is aligned to patient need (Ferguson, 2012), and has been shown to be critical for addressing low uptake and adherence (Kohl et al., 2013). Furthermore, this person-based approach maximizes opportunities for interventions to fully address the priorities and needs of the target population (Yardley et al., 2015).

Intrinsic motivation has previously been shown to be enhanced by positive feedback, but diminished by negative feedback (Deci, 1975). In the present study, the CBAT provided participants with trial-by-trial visual feedback for correct responses. In the open-ended questions however, five participants reported the worst aspect of their experience with the CBAT program was inconsistency in the trial-by-trial feedback they received, whereby they felt that the feedback did not reflect how they perceived they were performing. As such, it is possible that this may have affected their levels of intrinsic motivation. Ensuring consistency in the trial-by-trial feedback is therefore a key consideration for future CBAT development that aims to maximize intrinsic motivation.

#### Extrinsic motivation

The main theme contributing to participation in this study was participants' hearing difficulties. This provides a direct link between participants' health condition and their motivations for taking up the CBAT intervention. Furthermore, participants reported being influenced by the fact that the invitation to join the study was received via their family physician. In a recent examination of patient perceptions of benefit and enjoyment of auditory training, Tye-Murray and colleagues suggest that compliance with auditory training might be further enhanced if patients have regular contact with a hearing professional and train with meaning-based materials (Tye-Murray et al., 2012).

A substantial body of research has demonstrated that contexts that are supportive of autonomy, competence, and relatedness foster greater internalization and integration of behaviors, and therefore facilitate extrinsic motivation (Ryan and Deci, 2000). With this in mind, a number of recommendations can be made to increase extrinsic motivation in future CBAT design.

##### Autonomy

Within SDT, extrinsic motivation can demonstrate different levels of internalization. For example, individuals may either personally grasp that a CBAT intervention may offer benefit to address their hearing difficulties, and subsequently they adhere to the agreed intervention through personal endorsement (high internalization). Alternatively, individuals can be recommended or instructed to do the training, signaling compliance with external guidance (low internalization). In order to internalize behaviors, individuals must be able to relate to that meaning in terms of their own goals and values. Autonomy refers to choice and freedom from external pressure to behave in a certain way. Providing individuals with the freedom of task selection within CBAT interventions (i.e., the choice to select tasks that are most relevant to them and their hearing difficulties) may therefore help promote the personal importance of the intervention and increase conscious valuing (Tye-Murray et al., 2012).

##### Competence

People are more likely to adopt activities that they feel they can be effective in. So, any future CBAT development should bear this in mind. Ensuring that CBAT software is easy to use and achievable will help achieve and maintain usability and desirability.

##### Relatedness

Behaviors are reinforced when they are prompted, modeled, or valued by significant others. Within hearing rehabilitation there is growing evidence to suggest that the involvement of significant others offers additional benefit to individuals with hearing loss (Preminger, 2003; Pichora-Fuller et al., 2013). Furthermore, the significant other themselves may also gain benefit from their involvement (Pyykkö et al., 2014). Thus, the involvement of significant others in the delivery and ongoing support of CBAT interventions may serve to promote relatedness and increase motivation.

## Limitations

It should be noted that one of the researchers (HH) was involved in quantitative data collection for outcome measures at some participant tests sessions, and was also present in the two post-training focus groups. Although HH was not the primary facilitator, we are unable to rule out the possibility that the involvement of HH in both quantitative and qualitative data collection may have influenced the qualitative data for some participants.

A secondary theme from the focus groups accounting for engagement and adherence with CBAT was participants' desire to help other people with hearing loss by taking part in research. This was particularly true for those participants who had retired from work and had time to spare. As such, this is unlikely to be a factor associated with engagement and adherence with CBAT outside of a research environment. Participants who took part in the RCT and subsequent focus groups were a volunteer sample, and therefore may have different motivations than people with hearing loss who did not choose to take part. In addition, the nature of qualitative research means that findings cannot be universally applied to other populations. This study involved people with mild hearing loss who did not have hearing aids. It is possible that people with greater degrees of hearing loss or those who had already received an intervention (e.g., hearing aid users) may have different motivations for CBAT uptake, engagement and adherence.

Although informative in the short-term, the results of this research do not provide information about engagement and adherence to CBAT over time. One of the main intrinsic motivation factors associated with engagement and adherence in this study was that the training task was simple and easy to use. However, it is not clear from this investigation whether the simplistic nature of the task could lead to boredom or frustration over an extended period. It is also important to note that the training schedule in this study was substantially shorter than other CBAT programs such as LACE, and this may have contributed to the positive appraisal of the intervention, and to the high adherence rates witnessed in this study.

Participants in the RCT received weekly phone calls to identify any technical or procedural issues with the training software, which may or may not have contributed to the high training adherence witnessed in this study. Nevertheless, the telephone calls were not reported by participants to be a factor associated with training in either the post-training questionnaire responses or the focus groups.

As is the case with all research, the participants who took part in this investigation were volunteers. As such, it should be acknowledged that these individuals might be more motivated to engage with and adhere to the CBAT intervention than individuals in the general population. Furthermore, we cannot firmly rule out the effects of individuals' expectations regarding the benefits of the CBAT intervention. Finally, due to the high rates of adherence with CBAT witnessed in the RCT, the findings of this investigation cannot inform us about how we might best support adherence for individuals who may be less motivated to adhere.

## Summary and conclusions

Self-management of hearing loss requires motivation and dedication. Participants in this study readily perceived benefits of a 4-week CBAT intervention in terms of improved concentration and attention. Participants reported that the CBAT also made them more aware of their hearing difficulties, and prompted some individuals to seek further intervention (hearing aids) to address these difficulties.

Initial participation in the study (CBAT uptake) was associated with extrinsic motivation arising from participants' hearing difficulties, whereas engagement and adherence with CBAT was influenced by both intrinsic and extrinsic motivation including a desire to beat previous scores on the training task, and to help others with hearing loss.

The use of SDT as a theoretical framework to retrospectively interpret data in this investigation has provided useful insights into the applied nature of human motivation for CBAT. Furthermore, this approach offers a framework from which to develop future research to explicitly assess individuals' motivations for audiological intervention that maximize their intrinsic and extrinsic motivations for adherence. We see this as an important first-step in informing future theory-driven development of CBAT.

## Author contributions

MF designed the study. HH, AM, and MF analyzed and interpreted the data. HH and AM wrote the manuscript. MF and HH contributed to critical discussions. HH revised the manuscript. All authors approved the final version of the manuscript for publication. All authors agree to be accountable for all aspects of the work and in ensuring that questions related to the accuracy or integrity of any part of the work are appropriately investigated and resolved.

### Conflict of interest statement

The authors declare that the research was conducted in the absence of any commercial or financial relationships that could be construed as a potential conflict of interest.
